# Undertaking a scoping review: A practical guide for nursing and midwifery students, clinicians, researchers, and academics

**DOI:** 10.1111/jan.14743

**Published:** 2021-02-04

**Authors:** Danielle Pollock, Ellen L. Davies, Micah D. J. Peters, Andrea C. Tricco, Lyndsay Alexander, Patricia McInerney, Christina M. Godfrey, Hanan Khalil, Zachary Munn

**Affiliations:** ^1^ JBI Faculty of Health and Medical Sciences The University of Adelaide Adelaide SA Australia; ^2^ Adelaide Nursing School Faculty of Health and Medical Sciences The University of Adelaide Adelaide SA Australia; ^3^ Rosemary Bryant AO Research Centre Clinical & Health Sciences University of South Australia Adelaide SA Australia; ^4^ The Centre for Evidence‐based Practice South Australia (CEPSA): A JBI Centre of Excellence Adelaide SA Australia; ^5^ Li Ka Shing Knowledge Institute of St. Michael's Hospital Unity Health Toronto Toronto ON Canada; ^6^ Epidemiology Division and Institute of Health Management, Policy, and Evaluation Dalla Lana School of Public Health University of Toronto Toronto ON Canada; ^7^ Queen's Collaboration for Health Care Quality: A JBI Centre of Excellence Kingston ON Canada; ^8^ School of Health Sciences Robert Gordon University Aberdeen UK; ^9^ The Scottish Centre for Evidence‐based Multi‐professional Practice: A JBI Centre of Excellence Aberdeen UK; ^10^ Faculty of Health Sciences University of the Witwatersrand Johannesburg South Africa; ^11^ The Wits‐JBI Centre for Evidence‐Based Practice: A JBI Affiliated Group Johannesburg South Africa; ^12^ School of Nursing Queen's University Kingston ON Canada; ^13^ School of Psychology and Public Health Department of Public Health La Trobe University Melbourne Vic Australia

**Keywords:** evidence synthesis, methodology, midwifery, nursing, PRISMA‐ScR, reporting, scoping review

## Abstract

**Aim:**

The aim of this study is to discuss the available methodological resources and best‐practice guidelines for the development and completion of scoping reviews relevant to nursing and midwifery policy, practice, and research.

**Design:**

Discussion Paper.

**Data Sources:**

Scoping reviews that exemplify best practice are explored with reference to the recently updated JBI scoping review guide (2020) and the Preferred Reporting Items for Systematic Reviews and Meta‐Analyses Scoping Review extension (PRISMA‐ScR).

**Implications for nursing and midwifery:**

Scoping reviews are an increasingly common form of evidence synthesis. They are used to address broad research questions and to map evidence from a variety of sources. Scoping reviews are a useful form of evidence synthesis for those in nursing and midwifery and present opportunities for researchers to review a broad array of evidence and resources. However, scoping reviews still need to be conducted with rigour and transparency.

**Conclusion:**

This study provides guidance and advice for researchers and clinicians who are preparing to undertake an evidence synthesis and are considering a scoping review methodology in the field of nursing and midwifery.

**Impact:**

With the increasing popularity of scoping reviews, criticism of the rigour, transparency, and appropriateness of the methodology have been raised across multiple academic and clinical disciplines, including nursing and midwifery. This discussion paper provides a unique contribution by discussing each component of a scoping review, including: developing research questions and objectives; protocol development; developing eligibility criteria and the planned search approach; searching and selecting the evidence; extracting and analysing evidence; presenting results; and summarizing the evidence specifically for the fields of nursing and midwifery. Considerations for when to select this methodology and how to prepare a review for publication are also discussed. This approach is applied to the disciplines of nursing and midwifery to assist nursing and/or midwifery students, clinicians, researchers, and academics.

## 摘要

**目的:**

本研究旨在讨论可用的方法论资源和最佳实践指南, 以便确定和执行涉及护理和助产政策、实践及研究的范围审查。

**设计:**

讨论稿。

**数据来源:**

参考最近更新的JBI范围审查指南 (2020年) 以及系统审查和荟萃分析范围审查扩展的首选报告项目 (PRISMA‐ScR) , 以便探讨最佳实践的范围审查程序。

**护理和助产学启示:**

范围审查可用于合成证据, 目前越来越普遍。其主要用于解决大量研究问题, 并汇集通过不同来源获得的证据。范围审查是一种有用的证据合成方式, 适用于护理和助产程序, 并可为研究人员提供机会, 帮助其审查广泛的证据和资源。然而, 仍应严格执行范围审查程序, 且应保证其透明度。

**结论:**

本研究可为研究人员、临床医师提供指导和建议, 帮助其合成证据, 并在护理和助产领域应用范围审查方法。

**影响:**

伴随着范围审查日益普及, 多个学术和临床学科人员提出应保证方法的严谨性、透明度和适当性, 包括护理和助产学。本讨论稿的重点在于范围审查的各组成部分, 包括: 提出研究问题和目标; 制定方案; 确定资格标准和拟议搜索方法; 寻找和选择证据; 提取和分析证据; 展示结果以及概述护理和助产领域的具体证据。此外, 讨论内容包括方法选择时间和评论发表方法。此方法适用于护理和助产学科, 可用于协助护理和/或助产学生、临床医生、研究人员和学术人员。

## 1 INTRODUCTION

Scoping reviews are an invaluable form of evidence synthesis. Foundational concepts and evidence can be mapped, allowing for examination of practice, policy, and research and gaps in evidence and policy can be identified. The results of scoping reviews can provide indications for where further research may be required and inform the development of these research endeavours (Khalil et al., 2016; Munn, Peters, et al., 2018; Tricco et al., 2016). Scoping reviews have become increasingly popular, particularly in the health and social science disciplines, and they are broadly accepted as a helpful adjunct for informing new research projects (Pham et al., 2014; Tricco et al., 2016). As the popularity of scoping reviews has increased, so too have the criticisms of this methodological approach for synthesizing evidence (Davis et al., 2009; Tricco et al., 2016). Davis et al. (2009) undertook a review that explored the nature and status of scoping review studies in nursing literature. Their findings suggested that scoping reviews in the discipline were poorly understood and there was a lack of consistency and methodological rigour (Davis et al., 2009). Criticism of researchers' approaches to conducting scoping reviews is not limited to the field of nursing. Tricco et al. (2016) conducted a review of scoping reviews and found variability in approach among the 494 included reviews and highlighted the need for a standardized reporting guideline specific to the scoping review approach. The purpose of the current study is to highlight available methodological resources and best‐practice guidelines for the development and completion of scoping reviews relevant to nursing and midwifery practice and research.

## 2 BACKGROUND

The first framework for conducting a scoping review was proposed by Arksey and O'Malley (2005) and remains popular across disciplines (Pham et al., 2014). Extensions of this framework were later provided by Levac et al. (2010) in response to confusion and criticisms of the Arksey and O'Malley approach. These initial attempts have provided guidance to many researchers, but a lack of methodological clarity continues to exist, particularly with regards to the analysis of data. In response to ongoing concerns about the scoping review methodology, the JBI guidance for scoping reviews was developed by a working group of methodological experts (the Scoping Review Methodological working group). The aim in developing the guidance was to clarify each element required in a scoping review (Peters, Godfrey, et al., 2020). This guidance was developed through a consultative process with key stakeholders (Khalil et al., 2020; Peters, Godfrey, et al., 2020; Peters, Marnie, et al., 2020).

## 3 DATA SOURCES

Scoping reviews that exemplify best practice were explored with reference to the recently updated JBI scoping review guidance (Peters, Godfrey, et al., 2020; Peters, Marnie, et al., 2020) and Tricco, Lillie, et al. (2018) and Tricco, Zarin, et al. (2018) Preferred Reporting Items for Systematic Reviews and Meta‐Analyses Scoping Review extension (PRISMA‐ScR) (Tricco, Lillie, et al., 2018; Tricco, Zarin, et al., 2018). This was supported by the varied experience of the authors who comprise methodologists, researchers, and clinicians who share an interest in evidence‐based health care.

## 4 DISCUSSION

### 4.1 When should a scoping review methodology be selected?

When planning any research project, it is important to select the correct methodology. There are several approaches for conducting evidence synthesis (Grant & Booth, 2009; Munn et al., 2018). Each has its merits, but they are not all suitable for all research questions. Scoping reviews share some similar methodological principles as other types of evidence synthesis. For example, both scoping reviews and systematic reviews provide a synthesis of evidence to address a particular research question after a rigorous and systematic search of available literature (Peters, Godfrey, et al., 2020). The major differences between scoping and systematic reviews are the purposes for conducting these investigations and the intended use of the results (Peters, Godfrey, et al., 2020). A systematic review should be conducted if the intention is to produce evidence to inform decisions about feasibility, appropriateness, meaningfulness, or effectiveness of a particular treatment or practice (Munn, Peters, et al., 2018; Munn, Stern, et al., 2018). For example, these decisions could relate to the effectiveness of an intervention, prognosis of a condition, diagnostic accuracy of a test, and experiences of a phenomenon (Munn, Stern, et al., 2018). As such, systematic reviews will often inform policy decisions and clinical practice and may form the basis of trustworthy clinical guidelines. Scoping reviews map the literature and provide an overview of evidence, concepts, or studies in a particular field. Although scoping reviews may also be used to inform policy and practice, the type of decisions they inform are not necessarily related to questions of feasibility, appropriateness, or effectiveness, but more so around priorities for research, clarifying concepts and definitions, providing research frameworks or providing background, or contextual information on phenomena or concepts. Appropriate indications for scoping reviews are to identify knowledge gaps, scope a body of literature, clarify concepts, or to investigate research conduct (Munn, Peters, et al., 2018; Tricco, Lillie, et al., 2018; Tricco, Zarin, et al., 2018).

**FIGURE 1:**
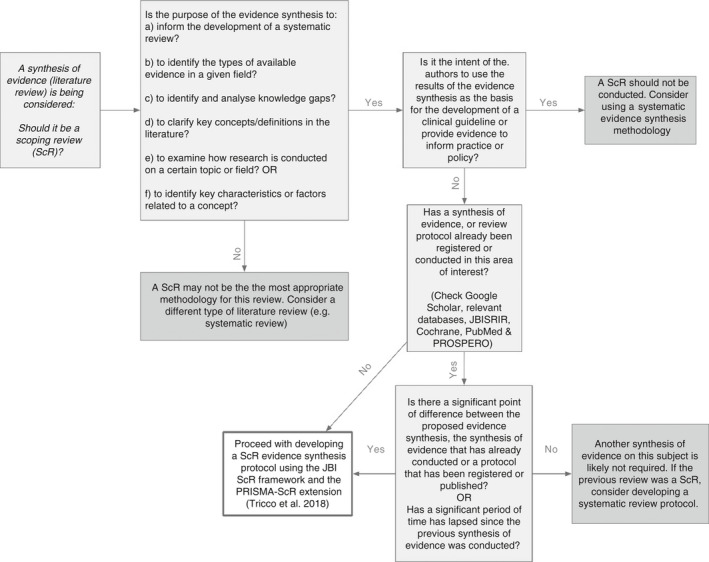
Considerations for selecting a scoping review methodology

Figure 1 provides an overview of the major considerations required for selecting a scoping review methodology. Of note, it is important to recognize that the role of a scoping review is not to provide recommendations for practice or to inform clinical guidelines. It is also not recommended that scoping reviews address questions about the experiences of populations unless it is designed as a preliminary search of literature that will inform the development of a systematic review. Reviews that seek to describe experiences or current practice will be more useful to the clinical and academic community if they are conducted using a systematic review methodology.

There are some additional considerations when planning to undertake a scoping review. These include available resources, such as databases and other potential sources of data (e.g., policies or practice frameworks), co‐authors for the study selection and extraction process, software to support the process (such as SUMARI and/or reference management software) (Munn et al., 2019), an academic librarian to assist with preparing the search strategy, and sufficient time (Peters, Godfrey, et al., 2020). It may also be prudent to consider where the protocol and final review may be published once they have been completed.

Other suggestions that may assist with preparing a scoping review for publication in a peer‐reviewed journal include:

1. Registering scoping review and/or develop a scoping review protocol to submit for peer‐review publication.
2. Find a suitable target journal and review author guidelines to ensure that they publish scoping reviews.
3. Demonstrate that the review has been rigorously undertaken and complies with the JBI 2020 guide and PRISMA ScR (Tricco, Lillie, et al., 2018; Tricco, Zarin, et al., 2018).

You may also consider contacting the target journal's editor and asking if the review that is being proposed would be considered for inclusion in their publication. This is not a guarantee but may save some time if they do not consider the article to be suitable.

### 4.2 JBI guidance

When the decision is made to pursue a scoping review, several choices can be made, including which guidelines to use. It is recommended that the JBI approach is followed, as it is, to date, the most rigorous and defined methodology. The JBI approach for scoping reviews contains nine steps (Peters, Godfrey, et al., 2020) and expands on the work of Arksey and O'Malley (2005) and Levac et al. (2010). Concept explanations and definition clarifications are included to enhance the quality and understanding of scoping review methodology. The following presents an overview of the guidelines in relation to the discipline of nursing and midwifery and includes examples of best practice.

### 4.3 Protocol development and registration

Scoping review protocols can be registered through Fig Share (https://figshare.com/) and Web of Science (webofknowledge.com), but not currently through PROSPERO. Examples of protocol templates can be found on JBI SUMARI (Munn et al., 2019) or through the JBI Manual for Evidence Synthesis (Chapter 11) (Peters, Godfrey, et al., 2020).

Journal requirements for a registered, a priori scoping review protocol vary. The JBI and Scoping Review Methodological working group highly recommend that one is undertaken and indeed, JBI Evidence Synthesis requires a previously published protocol before they will accept a completed scoping review. There are a range of nursing and medical journals, besides JBI Evidence Synthesis, which will accept scoping review protocols. These include the Journal of Advanced Nursing, Systematic Reviews, BMC Medical Research Methodology, and BMJ Open. The advantage of developing a scoping review protocol is that it minimizes the potential for ad hoc decision‐making that may reduce the methodological rigour of the scoping review. Changes made from the protocol to the final scoping review report are allowed but should be transparent and be reported in the final report. For example, Bobbette et al.'s (2020) scoping review addressed changes that have been made to their data extraction form since the protocol stage.

### 4.4 Consultation with stakeholders

Arksey and O'Malley (2005), Levac et al. (2010), and the JBI guidance (Peters, Godfrey, et al., 2020; Peters, Marnie, et al., 2020) offer differing perspectives on the importance of consultation with key stakeholders throughout scoping reviews. Arksey and O'Malley (2005) suggest it is an optional component, however, Levac et al. (2010) argued that it should be considered a required component. JBI recommends that consultation should occur with key stakeholders, information scientists, research librarians, and experts throughout the development of the protocol, execution, and dissemination of the evidence (Peters, Godfrey, et al., 2020; Peters, Marnie, et al., 2020).

Research librarians/information scientists play an important role in the process of conducting a scoping review. Ideally, they should be contacted during the development of the protocol to help define the search strategy and to identify relevant databases. As each database has a different search approach, research librarians/information scientists can also help ensure equivalence with each search. Their time and expertise should be acknowledged in the scoping review publication.

Consulting with researchers or content experts in the relevant field is important during the process of conducting a scoping review. This type of consultation can enhance the relevance of the research and ensure that the search strategy includes the appropriate terms. They may also be useful in finding resources that may not be identified through the searching of databases, grey literature, and references. For example, researchers may communicate with others in the field to ask if they have documents that could fit the inclusion criteria.

Other stakeholders may include patients and their informal caregivers, policymakers, government agencies, patient advocacy organizations, and healthcare providers (Cottrell et al., 2015). Cottrell et al. (2015) identified the following reasons stakeholders should be included when conducting evidence synthesis:

- To inform researchers about topics that are needed and relevant to the identified community, thus reducing research waste (Glasziou & Chalmers, 2018).
- To assist with refining the research question, clarifying definitions, reviewing the research, and providing a deeper understanding of the phenomenon and;
- To identify research gaps.

Acknowledging the involvement of stakeholder's involvement in any publications is required. This could either be in the acknowledgement section, or if they meet the necessary requirements, as an author of the scoping review.

### 4.5 Developing review objectives and questions

Arguably, one of the most important steps to consider when producing a scoping review is the development of the review question. Without a clear question, a scoping review will lack direction and coherence. The review question should be directly related to the overall objective of the review, it should be transparent, and located in the introduction section of the paper (Tricco, Lillie, et al., 2018; Tricco, Zarin, et al., 2018).

One benefit of scoping reviews is that the review question can be broader than those developed for systematic reviews. Examples of broad review questions that have been undertaken previously include: ‘What is known from the existing literature about succession planning in nursing education?’ (Phillips et al., 2019, p. 888), ‘What is the nature of the evidence relevant to the provision of mental health interventions by midwives?’ (Coates & Foureur, 2019, p. 391), and ‘How have former [intensive care unit] patients and their families been involved in critical care research and/or [quality improvement] projects’ (Bench et al., 2018, p. 218). These questions seek information and knowledge regarding subjects in niche and emerging areas of healthcare provision and research. The results of these studies could lead to a refined and more specific systematic review or could identify a paucity of research in that area of interest.

Numerous formats have been developed to guide the inclusion of information in a review question, but not all of these are suitable for scoping reviews. When developing a question for a scoping review, the recommended format is the ‘PCC’ mnemonic, where the *Population, Concept, and Context* are described (Peters, Godfrey, et al., 2020). Table 1 provides examples of well‐crafted review questions and objectives that follow this method. In both examples, the population, concept, and context are clearly identified, and a direct relationship can be observed between the review question and the review's objectives. The objectives provide a statement of what the authors seek to accomplish. Typically, objectives will describe what will be investigated, identified, explored, determined, or mapped. It is important that all objectives relate directly to the review question. If they do not, the overarching question(s) may not sufficiently represent the scope of the review and should be revised.

**TABLE 1:** Examples of review objectives and questions from scoping review protocols

Authors (year)	Objective(s)	Review question(s)	Population/participants	Concept	Context
Kao, Peters & Ooi (2017)	To *investigate* QoL questionnaires available to paediatric patients following tonsillectomies with or without adenoidectomies for chronic infections or SDB	What QoL questionnaires are available for paediatric patients following tonsillectomies with or without adenoidectomies for chronic infections or SDB?	Paediatric patients <16 years of age Undergoing tonsillectomy ± adenoidectomy for chronic tonsillitis or sleep‐disordered breathing	Questionnaires utilized to assess QoL in the target population and context	Settings where the target population undergo the procedure of interest and where QoL questionnaires are used.
Yu, Steenbeek, Macdonald, MacDonald & McKibbon (2019)	To *identify* the characteristics of Indigenous healing strategies in Canada and approaches to improving cultural relevance to local Indigenous contexts	What are the characteristics (e.g., guiding principles, main components, and human resources) of Indigenous healing strategies in Canada? What approaches have been used in research process [sic] to improve the cultural relevance to local Indigenous contexts?	First nations, Inuit, and Métis Indigenous peoples of Canada who self‐identify by other terms derived from their nations, traditional lands, or languages	Literature that describes an Indigenous healing strategy in Canada, including any attempt to promote health and healing	All service settings in Canada, including health, justice, child welfare, reconciliation, and education

Abbreviations: QoL, quality of life; ScR, scoping review; SDB, sleep‐disordered breathing.

### 4.6 Developing eligibility criteria

Eligibility criteria will dictate the papers that will be included in the review. If these criteria are too broad, the volume of included papers may be too cumbersome for one review. If these criteria are too narrow, there is a risk that no suitable papers will be located.

**FIGURE 2:**
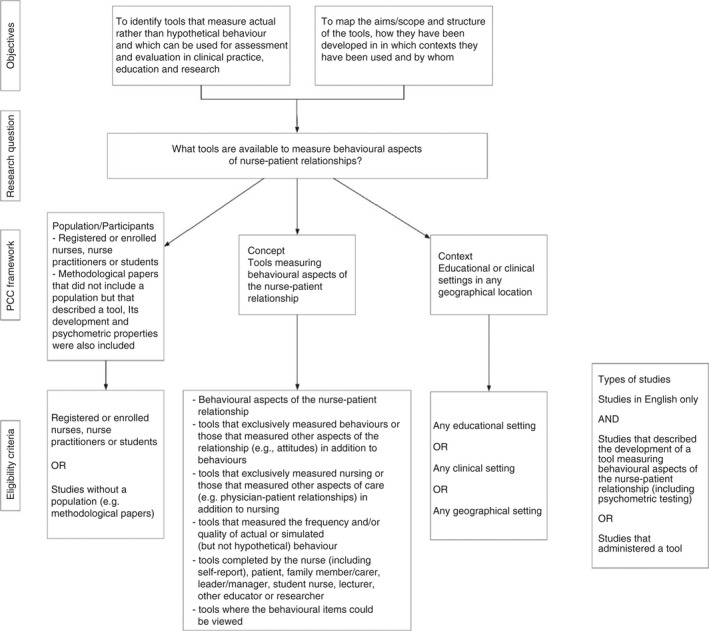
Relationship between research objectives, question(s) and eligibility criteria, Feo et al. (2020)

Eligibility criteria should be directly linked to the research objective(s) and question(s). The PCC framework used for developing the research objective(s) and question(s) will also inform inclusion and exclusion criteria and consequently the literature search strategy (Peters, Godfrey, et al., 2020; Peters, Marnie, et al., 2020). This is demonstrated well in Feo et al.'s (2020) scoping review and is outlined in Figure 2.

A rationale should be provided for all exclusion criteria (Tricco, Lillie, et al., 2018; Tricco, Zarin, et al., 2018). For example, if the review will be limited to a type of literature (peer‐reviewed articles) year of publication (within previous 10 years), geographical location (rural and remote settings), or population (individuals with Type 2 diabetes mellitus), a reason should be provided for why these limitations are required.

### 4.7 Describing the planned approach to evidence searching, selection, data extraction, and presentation of the evidence

Planning how the searching, selection, data extraction, and presentation of the evidence will occur needs to be documented in an a priori protocol. During planning stages, it is recommended that an Academic Librarian assists with developing the search strategy.

While a protocol is recommended, scoping reviews can be iterative and flexible. Concepts that may not have been discovered in the initial exploratory search may become a focus. If this occurs, changes to the protocol are permitted, but deviations need to be described in the final scoping review manuscript.

### 4.8 Searching for the evidence

The intention of this stage is to identify all relevant published and potentially unpublished evidence. Scoping reviews can include a broad scope of evidence, such as peer‐reviewed articles, news articles, theses, opinion pieces, and letters to editors. This information is not always located easily through a database search. Nonetheless, the search strategy must be reproducible and therefore the search process requires detailed documentation. Ideally, the search should be sensitive enough to identify all the relevant evidence, but specific enough that the search does not contain an excessive volume of irrelevant articles. Aromataris and Riitano (2014) describe how to develop a search strategy for systematic reviews, but this approach can also be applied to scoping reviews. The article describes the development of concept maps and logic grids that can provide visual representation of the search strategy and assist with identifying items relevant to your review (Aromataris & Riitano, 2014).

Searching for the evidence should occur in a broad range of relevant databases. For nursing and midwifery, these may include Medline, CINAHL, or OVID Emcare, Cochrane, Joanna Briggs Institute EBP, and Nursing and Allied Health databases. Further searches of clinical trial searchers, such as the Australian and New Zealand Clinical Trial Registry (ANZCTR), may be relevant. The PsycInfo database can also be useful for questions which combine nursing/midwifery practice with mental health, psychological, and social science concepts, for example, identifying the range of tools to measure behavioural aspects of the nurse–patient relationship (Feo et al., 2020). If the inclusion criteria contain theses, ProQuest Dissertation and Theses databases should also be searched.

A benefit of scoping reviews is the potential to include a variety of document types other than academic literature. This ‘grey literature’ is the information not controlled by traditional academic publishers and can include conference abstracts, theses, government reports, patents, and clinical practice guidelines, to name a few (Aromataris & Riitano, 2014). This is particularly useful in emerging fields, where peer‐reviewed publications may be limited, but other documents exist (Aromataris & Riitano, 2014). Grey literature can also be useful for understanding what resources are available to consumers, patients, or relatives. For example, Scott et al. (2019) explored guidelines which were easily accessible for handling storage of human breast milk through the search engines Google, Bing, and Yahoo, as well as Public Health sites. It may be valuable to include grey literature in a scoping review for a variety of reasons. For example, Gamble et al. (2020) included policy documents in their scoping review on hospital accreditation in midwifery care.

Aromataris and Riitano (2014) provide a detailed outline on how to search for grey literature, recommending resources such as OpenGrey.eu or Greylit.Org. The CADTH ‘Grey Matters: a practical tool for searching health‐related grey literature’ is also a useful tool in retrieving grey literature in a comprehensive and documented approach (https://www.cadth.ca/resources/finding‐evidence/grey‐matters; accessed 14 December 2020). Searching for grey literature can be difficult as it is not necessarily organized or indexed like peer‐reviewed articles in academic databases. Balancing the sensitivity and specificity of the search with resource limitations, particularly time restrictions, is challenging. If a grey literature search is conducted, it is necessary to determine and justify the extent of the search in the protocol and finalized scoping review.

Developing and implementing the search strategy should occur in three stages and in collaboration with a research librarian. These stages include:

1. *Initial search:* Search for articles relating to the review topic in at least two relevant databases and identify words and phrases found in the title, abstract, and index of papers that would likely be included in the review to inform your final search strategy
2. *Second search:* Using the identified search terms, formally conduct a search in the selected databases, and grey literature locations. Document these searches for inclusion in the final PRISMA flow chart (Tricco, Lillie, et al., 2018; Tricco, Zarin, et al., 2018).
3. *Reference list search:* Search the reference list of (a) all the identified studies from the initial search (consider time restrictions), (b) studies included from full‐text review, or (c) studies included in the review. It can also be useful to scan the reference list of related reviews identified in the search. To evaluate the search strategy, the Peer Review of Electronic Search Strategies (PRESS) is a checklist developed by librarians is a useful tool (Sampson et al., 2009; Sampson et al., 2008). During this stage, analyse the title of the articles and assess if it aligns with the review inclusion criteria. Details of how many studies were identified in the reference list search should be included in the PRISMA flow chart (Tricco, Lillie, et al., 2018; Tricco, Zarin, et al., 2018).

### 4.9 Selecting the evidence

Study selection is based on the eligibility criteria. Piloting the selection process, reviewing the management of disagreements, and the type of software that will be used in this stage need to be specified in the protocol and final scoping review manuscript.

#### 4.9.1 Piloting selection process

During each stage of evidence selection, at least two reviewers will review each article. Piloting this stage is important to ensure consistency across the review team. Developing an ‘elaboration document’, which provides details on each included and excluded document, can be helpful. There are various approaches to piloting source selection. The JBI Reviewers Manual for Scoping Reviews, for example, suggests each member reviews a sample of 25 titles/abstracts and then meets to discuss discrepancies and potential modifications. When agreement among team reaches 75% or greater, the selection of articles can continue (Peters, Godfrey, et al., 2020).

##### Managing disagreements

If there are disagreements between the two reviewers and consensus cannot occur, a third reviewer can assess the source to determine its eligibility.

##### Software for source selection

There are several applications suitable for assisting the selection of evidence. These include Covidence®, Endnote™, and Excel®. Peters (2017) has developed a step‐by‐step guide on managing source selection through endnote and has aligned this approach with PRISMA guidelines (Tricco, Lillie, et al., 2018; Tricco, Zarin, et al., 2018).

### 4.10 Extracting the evidence

Once sources have been selected for inclusion, evidence can be extracted. Two steps should occur before data are formally extracted. The first, during the protocol development stage, is to develop a standardized extraction form. Secondly, pilot testing of the form with two or more reviewers with two to three papers to ensure consistency. In scoping reviews, this may be an iterative process and the form may be adjusted. If the extraction form changes between the protocol stage and conducting the scoping review, it should be stated in the scoping review. An example of a data extraction table is provided in Table 2.

**TABLE 2:** Example of data extraction form for scoping reviews

Article title	Authors	Journal	Date of Publication	Population	Context	Concept	Methodology	Outcomes	Key Findings
									

#### 4.10.1 Critical appraisal or risk of bias

Critical appraisal and risk of bias assessments are not required in scoping reviews, however, some methodologists (Levac et al., 2010) do suggest that quality appraisal be considered. If critical appraisal or risk of bias is performed, an explanation for why it is being conducted should be outlined. The process and critical appraisal tools used also need to be described to improve transparency and methodological rigour.

### 4.11 Analysis of the evidence

The intention of scoping reviews is to provide a map and summary of available evidence, not to synthesize results into a set of final estimates or findings to inform decision‐making. Analysing the evidence gathered from the included studies is therefore normally descriptive, such as through frequency counting and basic coding. This can include organizing qualitative data into categories. An example of this type of qualitative descriptive approach can be seen in a scoping review that was investigating the needs of individuals recovering from a first episode of mental illness (Davies et al., 2018). The purpose of the review was, in part, to identify the needs experienced by individuals from this population. To facilitate a meaningful response to the review question, items of need identified in included articles were extracted and placed into categories (Davies et al., 2018).

It is common to see attempts to thematically analyse information in scoping reviews. This approach is not inherently wrong, but it does conflict with the purpose of scoping reviews: to map and chart the available evidence. If a review requirement is to examine or explore the experiences of a given population, then a qualitative systematic review may be more appropriate (Lockwood et al., 2015).

### 4.12 Presentation of the results

There are various approaches for presenting data from included articles. The selected approach should be described in the protocol and final scoping review manuscript. Commonly, scoping reviews use a tabular format to present the information gathered in the extraction and analysis stage. These tables should include the components of the PCC mnemonic and other relevant information, which aligns with the objectives and research question.

There are no defined rules on how to present results. Interesting examples include Fernandes Agreli et al.'s (2019) word cloud that was developed through NVivo to describe the most common words used to describe patient involvement in infection prevention and control guidelines. Kynoch et al. (2019) created a honeycomb heat map to provide a visual summary of the information needs and seeking behaviours of patients and families in acute healthcare settings. Other styles of presentations include pie charts and bubble plots. Alongside any visual representations, a narrative description is also required.

### 4.13 Summarizing the evidence

The discussion and conclusion paragraphs provide an opportunity to summarize evidence described in the included papers and to link this to the broader clinical and academic context (Peters, Godfrey, et al., 2020; Tricco, Lillie, et al., 2018; Tricco, Zarin, et al., 2018). When undertaking this task, it can be tempting to discuss issues that are tangential to the purpose of the review. Alignment between the summarized evidence of the review and the review question and objectives is vital for the cohesion of a review. As such, a few considerations may assist with compiling the discussion and conclusion sections.

Firstly, *have all elements of the review question(s) and objective(s) been addressed?* If all elements of the review question and objectives have been met in the results section, the discussion section can focus on the extent of evidence available and place this evidence into context. Given the nature of scoping reviews, there are circumstances where review questions and objectives may not be addressed due to insufficient literature. If this is the case, the discussion section provides an opportunity to discuss gaps in knowledge, new hypotheses, and considerations for future research.

Secondly, *has the review question been addressed accurately?* The discussion section provides an opportunity to demonstrate the alignment of review results with review questions and objectives. When conducting a scoping review, the information that is located can highlight new avenues of inquiry. It can be tempting to discuss these tangential subjects in the discussion section, without re‐focussing on the primary aim of the review. Ensuring alignment among the review question, objectives, results, and discussion will strengthen the integrity of the review.

Thirdly, *has the paper been adequately situated within the context of the relevant field of literature, practice and/or policy?* A good discussion section will highlight the contribution the review has made to the relevant field through reflecting on what has preceded the review and projecting the potential implications for future investigation and planning. The purpose of many scoping reviews is to describe the nature and diversity of available evidence (Peters, Godfrey, et al., 2020; Peters, Marnie, et al., 2020). As such, the discussion section should describe, with detail, the gaps in knowledge relating to the phenomenon, context, or concept that is under investigation.

The discussion will include a description of the strengths and limitations of the review. A significant strength of a scoping review will be the demonstration of compliance with a rigorous methodological and reporting framework. This can be achieved by transparently documenting the review process and adhering to the JBI (2020) guidance and the PRISMA‐ScR (Tricco, Lillie, et al., 2018; Tricco, Zarin, et al., 2018).

Review limitations that may be described can be divided into two broad categories: limitations relating to the methodology of the scoping review and limitations of the available research, literature, policy, and practice documents that were available to address the review questions and objectives. Limitations of the scoping review methodology include the absence of methodological and risk of bias evaluations and the resultant inappropriateness of the review to be used as evidence for clinical guidelines, limitations, and recommendations (Arksey & O'Malley, 2005; Peters, Godfrey, et al., 2020; Tricco et al., 2016).

*Implications for practice* is often a section that is requested by nursing and midwifery journals. As the purpose of a scoping review should not be to provide recommendations for clinical practice or policy change, this section can be challenging to compose. Some suggestions for addressing this section in a scoping review have been included in the JBI 2020 guidance (Peters, Marnie, et al., 2020). These include identifying gaps in knowledge identified in the review, describing specific implications for future research, and making suggestions for the conduct of a more specific research question that could be investigated through a systematic review (Peters, Godfrey, et al., 2020).

### 4.14 Using the PRISMA‐ScR

Scoping reviews are required to demonstrate the same transparency and reporting standards applied to systematic reviews. The original PRISMA was developed for systematic reviews and does not include some considerations relevant to scoping reviews. The PRISMA‐ScR contains 20 essential items, which should be reported and two optional items (critical appraisal of individual sources and within sources of evidence) (Tricco, Lillie, et al., 2018; Tricco, Zarin, et al., 2018). The PRISMA‐ScR is not to be used instead of the JBI guide (Peters, Godfrey, et al., 2020; Peters, Marnie, et al., 2020), but in conjunction. The JBI guidance provides a structure for how to initiate, develop, and undertake a scoping review; the PRISMA ScR is used to assist in developing a scoping review manuscript for publication to ensure it meets reporting standards (Tricco, Lillie, et al., 2018; Tricco, Zarin, et al., 2018).

### 4.15 Additional resources

Resources are available to assist with planning and developing scoping reviews. If there are challenges with deciding what type of review to undertake, the website: https://whatreviewisrightforyou.knowledgetranslation.net/ (accessed 12 December 2020), provides a useful decision‐making tool. Further resources include the JBI Scoping Review Working Group (scopingreviews.jbi.global; accessed 12 December 2020) and their newly updated JBI reviewer's manual (https://wiki.jbi.global/display/MANUAL/Chapter+11%3A+Scoping+reviews; accessed 12 December 2020). Another resource, which includes video presentations for the individual steps of a scoping review, is the UniSA Scoping Review website (https://guides.library.unisa.edu.au/ScopingReview; accessed 12 December 2020). Further information and resources about the PRISMA‐ScR can be found here: https://knowledgetranslation.net/portfolios/the‐prisma‐scr2/ (accessed 12 December 2020).

## 5 IMPLICATIONS FOR NURSING AND MIDWIFERY

Scoping reviews are a valuable form of evidence synthesis. The scoping review approach to evidence synthesis is increasingly being adopted by nurses and midwives who are seeking to map evidence and describe relevant literature. As the methodology for undertaking a scoping review advances and becomes more refined, it is important for nurses and midwives to be using the most current and appropriate guidelines, particularly if wanting to publish results or to use the results to inform future research. This study provides an overview of best practice and current guidelines for nursing and midwifery students, academics, and clinicians who are considering undertaking a scoping review. Examples and advice are offered to assist with the appropriate adoption of this methodology and the distribution of results to the broader community.

## 6 CONCLUSION

The scoping review methodology presents nursing and midwifery academics and clinicians with a valuable and adaptable opportunity to synthesize evidence. This approach to evidence synthesis has certainly grown in popularity in these professions and will no doubt continue to be used in the future. As this type of review continues to be adopted, it is vital that they are conducted rigorously and in accordance with the latest methodological recommendations. The process for how to perform a scoping review from inception to publication has been outlined in this study with a goal of facilitating conceptual clarity for nursing and midwifery academics, clinicians, and policymakers who are undertaking a scoping review.

### Peer Review

The peer review history for this article is available at https://publons.com/publon/10.1111/jan.14743.
